# A Rare Case of Pseudotumor Formation following Total Knee Arthroplasty

**DOI:** 10.5704/MOJ.1503.014

**Published:** 2015-03

**Authors:** S Sivananthan, R Pirapat, SB Goodman

**Affiliations:** Department of Orthopaedic Surgery, Stanford University School of Medicine, Redwood City, California

## Abstract

A 59 year old man who had undergone left total knee arthroplasty in 2008 presented with a 5 month history of left knee pain and persistent swelling. Workup for infection was negative and the patient was suspected to be suffering from particle disease and chronic synovitis. Imaging revealed an internally rotated tibial component. Intraoperative findings revealed extensive polyethylene wear with resultant metalon- metal articulation, soft tissue metallosis and a pseudotumor like mass at the posterolateral aspect of the popliteal fossa. This was extensively debrided and revision knee arthroplasty was performed. Suboptimal component alignment can lead to localized high loading, wear and metallosis, which in this case resulted in the formation of a pseudotumor.

## Case Report

A 59 year old man who had undergone left total knee replacement in November 2008 in another institution presented with a 5 month history of left knee pain and persistent swelling. He complained of a restriction in the range of movement and morning stiffness. The pain was at the front of the knee and medially more than laterally. It was worse with stairs, getting up from a chair, kneeling, squatting, getting into and out of a car and exercising. Sitting, medication and icing made the pain better.

Clinical examination revealed that the patient was 71 inches tall and weighed 285 pounds. There was a marked effusion in the left knee. It was slightly warm but there was no redness. The knee had a 10 degree fixed flexion contracture and could bend to 100 degrees. There was no instability in the antero-posterior or medio-lateral directions. The neurological and vascular examinations in the extremity were normal.

Past medical history included a successful right total knee replacement in 2007, spinal surgery in 1989, adrenalectomy in 2011 and prostate surgery in 2010. He also had a history of hypertension, prior hepatitis and dyslipidemia

**Fig. 1a-1d:**
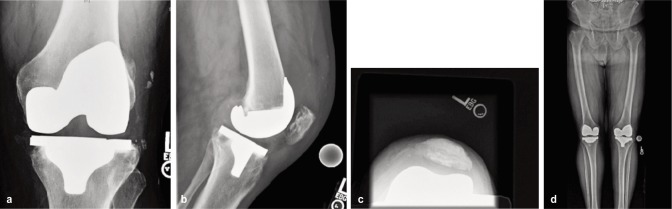
Preoperative views of Left Total Knee Replacement.

Radiographs showed an increase in patellofemoral joint space suggesting a marked effusion in the left knee and asymmetric medial joint space narrowing. There was the suggestion of internal rotation of the tibial component and periprosthetic lucency was noted in the posterior aspect of the medial and lateral tibial plateau, consistent with polyethylene wear and particle disease (Figure 1). CT scan showed internal rotation of the tibial component and joint effusion. The patient had a posterior stabilized Triathlon TM knee (Stryker Corp, Kalamazoo, MI). Both femoral and tibial knee components were made of cobalt chrome alloy, with a polyethylene spacer.

Pre operative Complete Blood Count (CBC) was within normal limits. Erythrocyte Sedimentation Rate (ESR) was slightly raised at 28mm/hr and C reactive protein (CRP) was slightly elevated at 5.7 mg/L. Aspiration of the knee revealed cloudy fluid and gram stain, cell count with differential and cultures were negative for infection. Three phase bone scan showed increased uptake on the blood pool phase consistent with synovitis, and increased uptake in the left knee on the delayed images.

Therefore revision knee surgery was performed. There was a florid synovitis and the polyethylene component was fractured and worn posterolaterally. There was a 2 ⨯ 3 cm area of significant metallic wear on the postero-lateral aspect of the femoral component. The metallic tibial component was also worn posterolaterally and internally rotated. It appeared that metal-on-metal wear was occurring in this area. There was an adjacent soft tissue mass which resembled a pseudotumor. The femoral component was well fixed to the bone and well positioned. However significant wear and shape change of the lateral femoral component was noted and this mandated femoral component removal. Debridement and synovectomy was performed followed by revision of the femoral and tibial components and the polyethylene insert. The patellar prosthesis was well fixed and not damaged. Therefore the original patellar component was left in place. The patient had delayed wound healing, and small areas were left to granulate in successfully. The patient had a successful post operative recovery and was mobilizing well at the 6 month follow up. The range of movement of the knee was 0 – 110 degrees and the knee was stable.

**Fig. 2:**
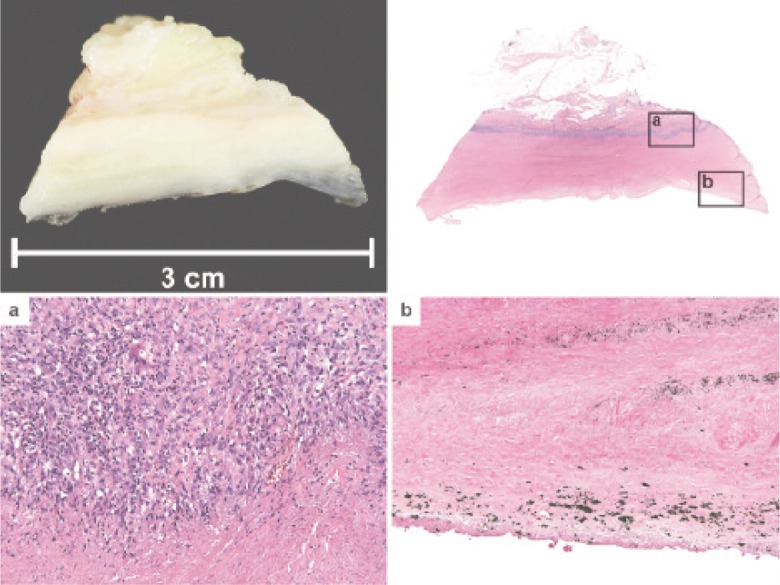
Gross specimen with low power magnification and high power magnification of relevant areas showing (a) granulomatous inflammation at the soft tissue interface and (b) pigmentation at the device interface.

Histology of the specimen revealed granulomatous inflammation, metallic and polyethylene wear debris, and areas of tissue necrosis and lymphocytic infiltration. (Figure 2) AFB and Gram staining were negative.

## Discussion

Wear and osteolysis is a leading cause for late reoperation in patients with total knee replacements (TKR). Five years after primary TKR, the main cause of revision is osteolysis due to polyethylene wear^3^. Osteolysis has been noted with both cemented and cementless modular TKRs, ranging between 6% and 30% for cementless TKR and between 0% and 16% for cemented TKR^2^. A review of literature revealed previous reports of pelvic, thigh or calf masses related to osteolysis and wear debris in patients with hip and knee joint replacements^2–6^. These masses have been reported up to 8 years following the primary joint replacement. In all cases, as in our case, the diagnosis was osteolysis and foreign body reaction caused by polyethylene wear and accumulation of metallic debris. The significant factors associated with implant wear are activity and weight of the patient, the type of articulation (constrained or non-constrained), the design of the implant, the bearing surfaces, the alignment and dynamic balancing of the prosthetic joint, and the thickness, the quality and the methods of sterilization of the polyethylene insert. Any combination of these factors may result in the generation of significant quantities of particulate debris. Concomitant metallosis leads to a vicious cycle of failure with increasing abrasive wear of the articulation. In our patient, the right knee mass consisted of a chronic inflammatory reaction to polyethylene wear debris and metallic particles. Intraoperative findings showed massive metallosis in the soft tissues around the tibial prosthesis, and periprosthetic osteolysis secondary to extensive wear, delamination and deformation of the polyethylene insert. Wear debris from implanted prostheses is generated by wear due to abrasion, adhesion and fatigue. In vitro studies have shown that macrophages exposed to wear particles produce cytokines that induce bone resorption. Prosthetic loosening leads to increased micromotion at the component interface, which then generates further particulate debris. Haynes6 described that Co-Cr particles cause early death of the macrophages, diminishing the inflammatory mediators implicated in osteolysis, whereas titanium particles were minimally toxic to the macrophage, thereby triggering higher levels of pro-inflammatory mediators. Cobalt chromium byproducts are therefore toxic to cells and result in cell death and lymphocytic infiltrate as seen in the histology in this case.

In the present case, the pseudotumor like mass emerged at the posterior-lateral aspect of the patient's prosthetic knee joint approximately four years after a total knee replacement. The mass consisted of a granulomatous inflammatory reaction to plastic and metallic wear particles, mimicking a pseudotumor mass like lesion in lateral popliteal fossa. Suboptimal component alignment associated with localized loading, polyethylene and metallic wear resulted in the formation of a pseudotumor.
